# Experimental-numerical Investigation of ⍺/β-phase formation within thin electron beam melted Ti–6Al–4V

**DOI:** 10.1016/j.heliyon.2024.e25971

**Published:** 2024-02-09

**Authors:** Garrett M. Kelley, M. Ramulu

**Affiliations:** a Department of Mechanical Engineering, University of Washington-Seattle, WA, 98195, USA; b Department of Materials Science and Engineering, University of Washington-Seattle, WA, 98195, USA

**Keywords:** Phase field method, Ti–6Al–4V, Electron beam melting, CALPHAD, Finite element method, Microstructure formation

## Abstract

Electron beam melting is a powder bed fusion process capable of manufacturing thin structural features. However, as the thickness of these features approaches typical microstructure grain sizes, it becomes vital to understand how the manufacturing process contributes to local crystallographic texture and anisotropy in micromechanical response. Therefore, this article investigates Ti–6Al–4V ⍺/β-phase formation within thin components using a variety of experimental and numerical approaches. Optical and scanning electron microscopy are used to determine through-thickness distributions of prior-β width ([top, middle, bottom]:[81.2 ± 44.2, 76.02 ± 30.4, 75.6 ± 31.2] μm), ⍺-lath thickness ([top, middle, bottom]:[1.0 ± 1.3, 1.3 ± 1.2, 1.4 ± 1.8] μm; average), and ⍺/β-phase fractions ([top, middle, bottom]:[0.87 ± 0.05, 0.82 ± 0.03, 0.88 ± 0.03]; average). Manufacturing process (i.e., “logfile”) data is used within a layer-by-layer finite element “birth/death” model. This model is loosely coupled with the Kim-Kim-Suzuki phase field model and a CALPHAD thermodynamic database to predict ⍺-lath growth throughout the process. In general, good correlation is found between the experimental data and the predicted temperature history, ⍺-lath coarsening, and phase fraction. This indicates that these tools would be useful in predicting process-structure-properties-performance relationships for thin features.

## 1 Introduction

Electron beam melting (EBM) is an additive manufacturing (AM) process that utilizes a fine metallic powder as the feedstock material. The feedstock diameter ranges from 40 to 120 μm in diameter and is typically deposited with a nominal layer thickness of 50 μm (i.e., about 50% the thickness of a sheet of paper). Given this layer resolution, EBM is applicable to parts with complex structural features that are a few millimeters in size. However, at these scales, it becomes necessary to understand how the process affects (as well as can be used to tailor) the local microstructure and hence mechanical response.

The focus of this article is on the Ti–6Al–4V alloy which is used extensively in the aerospace and biomedical industries. During solidification from melt, the high-temperature β-phase (BCC_A2_) forms prior (i.e., “prior-β”) to the nucleation and growth of the ⍺-phase (HCP_A3_) below the β-transus temperature, $T_{\beta }$ (≈1110K). This ⍺-phase nucleates along the prior-β grain boundaries and throughout the prior-β grains until a lamellar structure of ⍺ “laths” or platelets and β “ribs” (in between the platelets) develop. The relative size and orientation distributions of the two phases (i.e., “crystallographic texture”) directly affect the micromechanical behavior, leading to anisotropic elastic and plastic response. This is especially true for parts with structural features that approach the scale of the prior-β grains which are known to grow epitaxially with dominant columnar structures parallel to the direction of the build (i.e. +Z-direction) due to the higher temperature gradients and lower solidification velocities [[1], [2], [3]].

Similarly, the size of the ⍺-phase is dependent on the local temperature and cooling rate from the β-transus temperature. In general, 12 variants or orientations of the ⍺-phase can form that are a function of the relative orientation of the prior-β phase (i.e., the Burgers orientation relationship) and the local stress state (i.e., transformation strains due to crystal lattice mismatch). This results in a directional dependence of the ⍺-phase within the build plane (i.e., XY-plane) whereby the HCP unit cell results in an anisotropic mechanical response parallel or perpendicular to the basal plane. Therefore, understanding how the EBM process affects formation of the dominant crystallographic directions, as well as how those dominant directions affect the micromechanical response of thin structural features, is critical to engineering design and analysis.

To study how the build process affects the microstructural evolution of the as-built part, several models have been developed. For instance, Radhakrishnan developed a large-scale phase field model which incorporated free energy contributions due to interfacial and strain energies which demonstrated the evolution of multiple ⍺ variants. The model demonstrated that layer “bands” are developed (approximately three layers thick) below the heat source due to multiple heating and cooling cycles which straddle the ⍺-β transition temperature. Within this band, the microstructure is predominantly equiaxed/colony structured prior to formation of the basket-weave structure outside of the layer bands where the local temperature does not exceed the β-transus and dissociation of the ⍺-phase does not occur [4].

More recently, Xiang et al. developed a phase field model that investigated diffusionless, martensitic transformation of Ti–6Al–4V. To do this, they considered the time-dependent Ginzburg-Landau (or Allen-Cahn) equation which models the spatial and temporal evolution of non-conserved order parameters. In this case, the order parameters were selected as the six independent variants of the HCP ⍺-phase relative to the local BCC β-phase. In addition to the gradient free energy at the diffuse interface between the phases, the free energy formulation considered the local chemical free energy modeled using the Gibbs free energy difference between the ⍺- and β-phases as well as the stress-free transformation strain due to the lattice mismatch between the phases. Langevin noise was also used to describe the local thermal fluctuation in the ⍺-phase order parameters during nucleation. The author's found a preference for the formation of variant clusters with triangular morphology which can contribute to micromechanical behavior during void formation and crack propagation. These results were found to be consistent with microstructures measured in the SLM process which have higher cooling rates in comparison to EBM (due to their lower build chamber temperatures) which can result in diffusionless microstructural transformations [5].

Since the performance of an as-built component is a function of its microstructure, we should like to develop an understanding of how the EBM process affects microstructure evolution. While microscale numerical models such as the discrete element method (DEM) and computational fluid dynamics (CFD) are useful for understanding the interactions that occur at the particle scale, they are intractable at the mesoscales at which EBM occurs. In order to bridge these scales, a two-dimensional (2D) finite element model was developed based on element birth (activation)/death (deactivation) to investigate the temperature evolution that occurs during the layer-by-layer build process. To investigate ⍺-phase growth kinetics, a one-dimensional (1D) phase field model [6] was coupled to a Ti–6Al–4V thermodynamic database [7,8] using the CALPHAD approach [9,10]. The results for the models were compared to experimental data collected using optical and scanning electron microscopy. The purpose of this work was to investigate and validate the application of numerical models in predicting microstructural formation of thin structures.

## 2 Materials and methods

### 2.1 As-Built (step-ramp) specimens

**Fig. 1:**
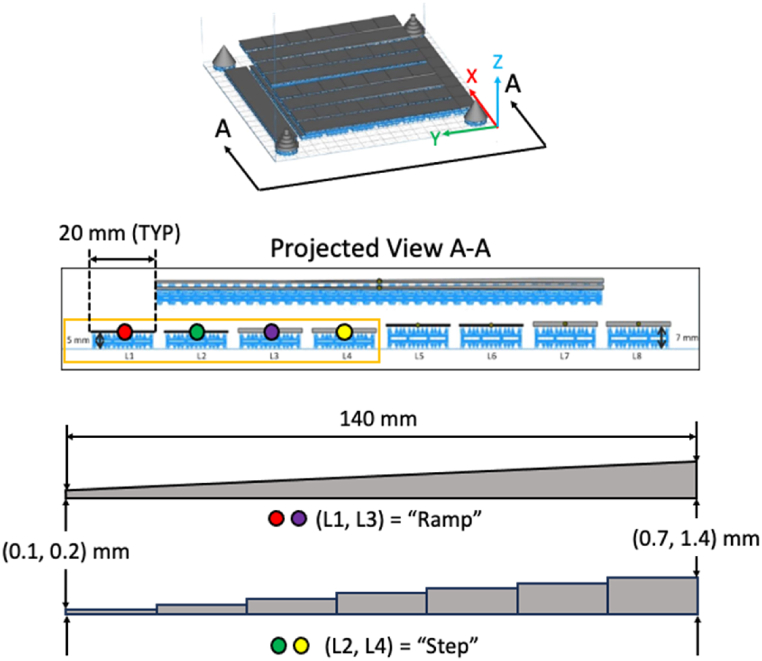
Overview of the as-built “ramped” (L1, L3) and “stepped” (L2, L4) specimens.

The four (4) specimens considered in this study were manufactured from Ti–6Al–4V powder feedstock subjected to five (5) reuse cycles while flushing with unused (i.e., “virgin”) powder. The Arcam A2X EBM machine and manufacturer-provided build parameters were used to manufacture each specimen [11]. Each specimen was nominally 20 mm wide and 140 mm long. Specimens L1 and L3 (i.e., odd numbered) had thicknesses that varied continuously along their length (i.e., “ramped”) from 0.1 mm to 0.7 mm, or 0.2–1.4 mm, respectively. Likewise, specimens L2 and L4 (i.e., even numbered) had thicknesses that varied discontinuously (i.e., “stepped”) with seven (7) steps (0.1 mm wide) from 0.1 mm to 0.7 mm, or 0.2–1.4 mm, respectively. At a nominal layer thickness of 50 μm, this results in 2–14 layers for the thinner specimens or 4 to 28 layers for the thicker specimens. For reference, the default Arcam A2X build parameters used to manufacture the specimens are provided in Table 1. Also, an overview of the as-built specimens is provided in Fig. 1.

**Table 1:** Default Arcam A2X build parameters for EBM of Ti–6Al–4V. Note that the parameters continuously vary over duration of the EBM manufacturing process.

***Build Parameter***	***Value***	***Units***
Beam Voltage	60	kV
Beam Speed	4530	mm/s
Speed Factor	1.5	–
Beam Current	15	mA
Max Current	20	mA
Focus Offset	25	mA
Line Order	1	–
Line Offset	0.2	mm
Hatch Depth	0.05	mm
Thickness Factor	0	–

#### 2.1.1 Specimen preparation

Each specimen was carefully sectioned length-wise along its centerline using a bandsaw (Roll-In Saw JM1220). Next, the halves were sectioned width-wise with a low-speed saw and wafering blade (Buehler IsoMet Low Speed) at 9 revolutions per minute (RPM) using coolant. Each section was mounted in graphite-based powder (Allied High Tech Products, Inc. 155–20010) according to the required cure schedule using a Buehler Pneumet I pneumatic hot mounting press. After mounting, each sample was wet sanded with 120, 240, 400, 600, and 800 grit silicon carbide (SiC) sandpaper at 20 N and 300 RPM for 3 min (Buehler EcoMet 30). A secondary polish was performed using a diamond suspension of 6 μm (DiaLube Suspension 90-3DL9) with a wool pad (DiaMat 90–150) for 5 min. A final chemical attack polish was performed using 10 mL of 0.05 μm colloidal silica, 0.5 mL of ammonium hydroxide (NH_4_OH), and 2 drops of hydrogen peroxide (H_2_O_2_) for 5 min. After each polishing step, the sample was ultrasonically cleaned with deionized water. Kroll's reagent (2% hydrofluoric acid (HF), 6% nitric acid (HNO_3_), and 92% water [H_2_O]) was applied for 8 s to etch the grain boundaries.

#### 2.1.2 Prior-β grain widths; ⍺ lath thicknesses; phase fraction

**Fig. 2:**
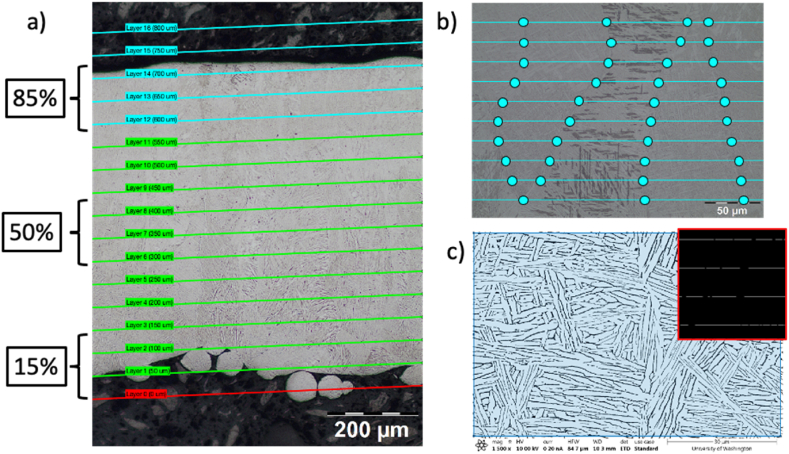
a) Approximate locations where optical micrographs are taken. b) ASTM E112 line intercept method used to calculate the width of the prior-β grains. c) SEM image of the ⍺/β microstructure after binarizing.

Characterization of the Ti–6Al–4V microstructure involves measuring the prior-β grain widths and ⍺-phase lath thicknesses. Imaging was performed using optical microscopy (OM; Nikon Eclipse LV150 N) and scanning electron microscopy (SEM; JEOL JSM-6010PLUS/LA). Optical micrographs were taken of the L1 specimen (thicknesses: 0.15 mm, 0.25 mm, 0.30 mm, 0.35 mm, 0.55 mm, and 0.70 mm) at regions near the bottom, middle, and top of the specimen which correspond to approximately 15%, 50%, and 85% from the bottom of each specimen as shown in Fig. 2a. This data was used to measure the prior-β grain widths. Similarly, scanning electron micrographs were taken in the same regions for specimens L1 (thicknesses: 0.10 mm and 0.50 mm; middle), L2 (thicknesses: 0.10 mm and 0.50 mm; middle), L3 (thicknesses: 1.4 mm; top and middle), and L4 (thicknesses: 0.95 mm, [top, middle, lower] 1.4 mm [lower]) and were used to measure the ⍺-lath thicknesses.

The prior-β grain widths were measured from the optical micrographs using the line intercept method from ASTM E112 [12] and a custom MATLAB script. In general, the grain widths were analyzed using ten (10) lines constructed perpendicular to the build direction. At each consecutive intersection between the lines and the prior-β grain boundaries, the length of the prior-β grain was calculated. An example of this is shown in Fig. 2b.

An additional MATLAB script was written to measure the ⍺-lath thicknesses using a procedure similar to that outlined by Searles et al. [13,14]. In general, prior to grayscale thresholding, the sharpness, contrast, and brightness of the SEM images were adjusted to decrease image noise and improve the color separation between the α- and β-phases. Binarizing was then performed to give a Boolean image, $I_{b}\text{,}$ with a value of ‘1’ representing the alpha phase and ‘0’ representing the β-phase. Parallel lines spaced 5 μm apart were then constructed at specific angles from the horizontal axis of the SEM image (i.e., 0°–150° in 30° increments; $L_{i}$). Using the Boolean image as a mask for the parallel lines (e.g., $I_{b}⨀L_{i}$ where ⨀ represents the element-wise multiplication operator) results in a population of fragmented lines where their lengths (collected using the ‘regionprops2’ function) represent the local ⍺-lath thickness. After distributions for the ⍺-lath thicknesses were collected, the average thickness was calculated after fitting the data to a Gamma distribution which has a minimum of zero. An example measurement is shown in Fig. 2c.

Finally, from the previous definition for the Boolean image, $I_{b}$, the phase fraction, $\varphi_{f}$, was calculated using the relationship provided in Equation (1):

(1) $$\varphi_{f}=\frac{N_{\alpha }}{N_{t}}$$

where $N_{\alpha }$ is the number of pixels representing the ⍺-phase and $N_{t}$ is the total number of pixels in the image.

#### 2.1.3 Specimen thickness and beam penetration depth

To understand how total specimen thickness correlated to both the nominal computer-aided design (CAD) geometry as well as the nominal layer thickness of 50 μm, the optical micrographs for the L1 specimen were used. A MATLAB script approximates the bottom “datum” layer with a single user-defined “best-fit” line. Next, 50 μm offsets parallel to this line were constructed until the top of the specimen in the optical micrograph was reached. An example of this measurement is shown in Fig. 2a where the datum layer (0 μm) is constructed in red while the nominal (“as-designed”) specimen thickness is outlined in green. Additional layers that are outside of the nominal specimen thickness are shown in blue. Note that these additional layers also serve as a crude estimate of the beam penetration depth.

### 2.2 Numerical models

#### 2.2.1 Layer-by-layer model

To study the thermal history that accompanies the layer-by-layer build process, a finite element model was built in Idaho National Laboratory's Multiphysics Object-Oriented Simulation Environment (MOOSE) [15] framework based on the element birth (activation) and death (deactivation) model. For this model, three different finite element subdomains are defined which correspond to the active (e.g., powder and molten/solidified material) and the inactive (e.g., vacuum) domains. Here, it is implied that the partial differential equations that model the physics are not solved on the inactive domain and that the material properties of the powder and the molten/solidified material are different. The analysis of the build process considers a two-dimensional (2D) domain 12.8 × 6.4 mm and the layer-by-layer construction of a 6.4 mm × 0.7 mm (i.e., equivalent to the thickest section of the L1 and L2 specimens). The initial layers are offset from the build plate by 5 mm which reflects the build setup [11]. The domain is meshed uniformly with quadrilateral elements 50 μm in size. The element sizes were selected for convenience based on the nominal layer thickness and to reduce computational expense.

To describe the evolution of the temperature field, T, during the build process, the energy equation provided by Equation (2) is solved:

(2) $$\rho c_{p}\frac{\partial T}{\partial t}-\nabla \cdot \left(k_{c}\nabla T\right)=\sigma_{sb}\epsilon \left(T_{\infty }^{4}-T^{4}\right)+{\dot{Q}}_{evap}+{\dot{Q}}_{b}$$

The first term on the left-hand side of the equation represents the change in internal energy of the material where ρ is the material density and $c_{p}$ is the specific heat (i.e., 4430 kg-m^−3^; 526.3 J-kg^−1^-K^−1^). The second term then represents conductive heat transfer with $k_{c}$ representing the thermal conductivity of the material (i.e., 22 W-m^−2^-K^−1^). The first and second terms on the right-hand side of the equation represent the radiative and evaporative heat transfer which are defined as fluxes (i.e., Neumann boundary conditions) on the moving interface defined by the deposited layers. The constants $\sigma_{sb}$ and ϵ represent the Stefan-Boltzmann constant and emissivity of the material, (i.e., 0.6) respectively, while $T_{\infty }$ is the ambient temperature taken to be the heat shield temperature of 640 K [16]. The evaporative heat transfer is calculated using the methods outlined in Refs. [17,18]. However, fluid dynamics of the melt pool are not considered to reduce computational cost. Since the goal of this model is to describe the temperature evolution of the layer-by-layer process, and the domain considered is much larger than the melt pool, this is an acceptable approximation. Finally, the remaining boundary conditions are Dirichlet boundary conditions equal to the preheat temperature (i.e., $T_{preheat}=973\;K$).

Continuing, the heat source was modeled as outlined by Zah and Lutzmann in Ref. [19]. Similar to the Klassen penetration model [20], this model defines the volumetric energy distribution of the beam as the superposition of two separate distributions as detailed in Equation (3) and Equation (4):

(3) $$I_{xy}=\frac{4\;\ln\left(0.1\right)}{\pi D_{b}^{2}d_{p}}\exp\left(4\;\ln\left(0.1\right){\left(\frac{x-v_{b}t}{D_{b}}\right)}^{2}\right)$$

(4) $$I_{z}=-3{\left(\frac{z}{d_{p}}\right)}^{2}+2\left(\frac{z}{d_{p}}\right)+1\;\forall \;\left\{z:h_{z}-d_{p}\leq z\leq h_{z}\right\}$$

where z is the absolute height of the beam that corresponds to the current layer height above the build plate and $d_{p}$ is the beam penetration depth, assumed to be 150 μm (or 3 layers). Note however that $I_{z}$ takes on values beyond three (3) layers. Therefore, $I_{z}$ requires the additional constraint on z provided. This model was chosen as opposed to Klassen's model because it allows for larger elements to be used. For instance, the penetration depth for Klassen's model was predicted to be approximately 18 μm which is smaller than the average element size of 50 μm [17,20].

**Fig. 3:**
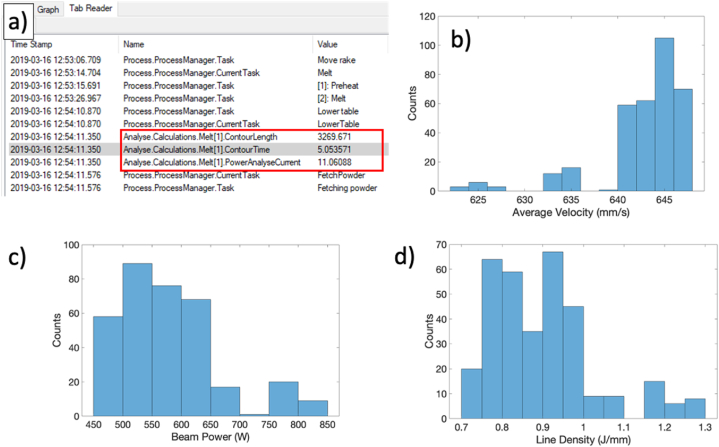
a) Example of the contour length, contour time, and beam current extracted from the logfile for the current layer at a given timestamp. Line density distribution (d) calculated from estimated average velocity (b) and beam power (c).

In general, solution of the finite element model occurs in three steps: 1) layer deposition; 2) beam scanning; and 3) cooldown. During layer deposition, the vacuum elements are switched to active and assigned the powder material properties at a rate equal to the Arcam A2X rake speed (i.e., 100 mm/s). For each layer, the direction of raking is switched. After deposition, the beam is scanned over the freshly deposited powder bed at a fixed beam power and scan speed (i.e., 660 W; 650 mm/s). These values were selected by examining the distribution of line densities as calculated using Equation (5):

(5) $$E_{L}=\frac{P_{b}}{v_{b}}=\frac{V_{b}i_{b}}{v_{b}}$$

for the entire build where $P_{b}$ represents the beam power, calculated using the beam acceleration voltage, $V_{b}$, (assumed to be 60000 V); the beam current, $i_{b}$; and the scanning speed, $v_{b}$. Estimation of the beam speed is accomplished by examining the Analyse.Calculations.Melt [1].ContourLength and Analyse.Calculations.Melt [1].ContourTime parameters in the Arcam A2X logfile for the Step-Ramp build which are used to calculate an average speed as given by Equation (6):

(6) $${\bar{v}}_{b}=\frac{L_{c}}{t_{c}}$$

where $L_{c}$ and $t_{c}$ are the given logfile contour lengths and times for the current layer, respectively. An example of this information is highlighted in Fig. 3a while Fig. 3b through Fig. 3d show the distributions for estimated contour speed, beam power, and calculated line density, respectively.

**Fig. 4:**
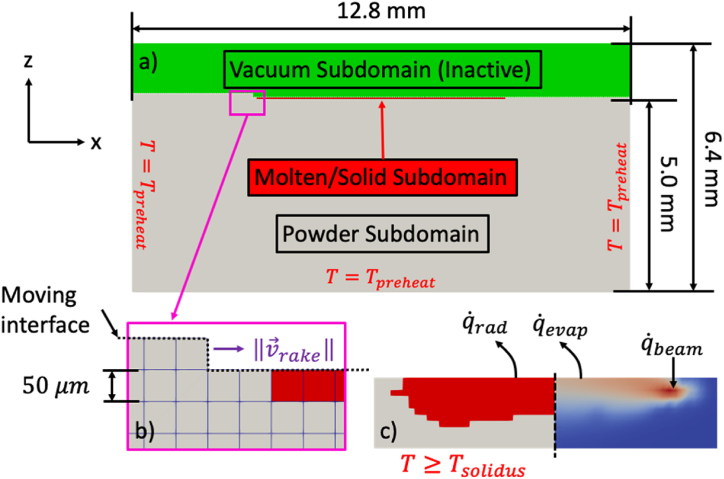
a) Overview of the computational domain composed of three separate subdomains: 1) Powder; 2) Molten/Solid; and 3) Vacuum (inactive). Also shown are the layer deposition (b) and beam scanning (c) steps.

If the local temperature of the powder exceeds the solidus temperature for Ti–6Al–4V (e.g., $T_{solidus}=1878\;K$), the powder material properties, assumed to be 60% of the molten/solid properties, are changed to the molten or solid material properties. To prevent local overheating of the material, a cooldown step was implemented whereby both the rake and the beam are switched off. The length of this cooldown phase was selected after examining the Arcam A2X logfile for the Step-Ramp build (i.e., the average time between “Melt” process steps was found to be 8 min). Initial numerical experiments showed that the temperature of the computational domain after beam scanning reached within a few Kelvin of the preheat temperature after 100X of the time scale of beam scanning which is about 1 s (i.e., $\frac{L_{c}}{v_{b}}=\frac{6.4}{650}\approx 0.01\;s$). Therefore, for solver stability, this was selected as the duration of the cooldown step. Given that the three steps occur over different time scales, MOOSE functions were used to control both time intervals and solver timestep size. After the cooldown step, the process was repeated. The three steps are outlined in Fig. 4 where Fig. 4a shows an overview of the computational domain while Fig. 4b and c show the layer deposition and scanning steps, respectively.

#### 2.2.2 Phase field model

The one-dimensional (1D) Kim-Kim-Suzuki (KKS) phase field model [6] is used to describe the β→⍺/β growth kinetics after solidification. For this model, we define a non-conserved order parameter, $\eta^{op}\left(x_{i},t\right)=\eta^{op}$, that describes the evolution of an arbitrary ⍺-phase variant relative to the parent prior-β phase where $\eta^{op}$ varies smoothly between 1 inside of the variant to 0 outside the variant. The formation and growth of the variant is governed by the following global free energy defined by Equation (7) [6,21,22]:

(7) $$F^{\beta \to \alpha /\beta }=\int_{\Omega }\left(f^{\mathrm{int}}+f^{chem}\right)\partial \Omega$$

where $f^{\mathrm{int}}$ and $f^{chem}$ are the interfacial and chemical free energy densities, respectively. The interfacial energy density is defined by Equation (8) [21]:

(8) $$f^{\mathrm{int}}=W{\eta^{op}}^{2}\cdot {\left(1-\eta^{op}\right)}^{2}+\frac{K_{\eta }}{2}{\left(\nabla \eta^{op}\right)}^{2}$$

where the first term accounts for the thermodynamic activation barrier and represents the energy required to create a new ⍺-phase interface within the parent β-phase. The magnitude of this activation barrier is represented by the coefficient W (units of $E/L^{3}$). Since the phase field method approximates the interface between the phases as diffuse (as opposed to sharp), the second term represents the energy penalty associated with this approximation where $K_{\eta }$ is the gradient energy coefficient (units of E/L). The benefit of the KKS model is that the diffuse interface width (0.2 μm), $\lambda_{w}$, can be selected independent of the activation barrier and gradient energy coefficient. The interfacial free energy (0.1 J-m^−2^), $\sigma_{\mathrm{int}}$, between the ⍺- and β-phases is related to the activation barrier (i.e., W), the gradient energy coefficient (i.e., $K_{\eta }$), and the interface width (i.e., $\lambda_{w})$ through the relationships provided in Equation (9) and Equation (10) [6]:

(9) $$\sigma_{\mathrm{int}}=\frac{K_{\eta }\sqrt{W}}{3\sqrt{2}}$$

(10) $$2\lambda_{w}=\frac{\alpha_{KKS}K_{\eta }\sqrt{2}}{\sqrt{W}}$$

where $\alpha_{KKS}$ is a constant that defines an interface width cutoff and is assumed to be 2.2 per [6].

Continuing, the chemical free energy density is defined by Equation (11) [21]:

(11) $$f^{chem}=\left(1-h\left(\eta^{op}\right)\right)\frac{G^{\beta }\left(X^{Al,\beta },X^{V,\beta },T\right)}{V_{m}}+h\left(\eta^{op}\right)\frac{G^{\alpha }\left(X^{Al,\alpha },X^{V,\alpha },T\right)}{V_{m}}$$

where $h\left(\eta^{op}\right)=\eta^{op}\left(6{\left(\eta^{op}\right)}^{2}-15\eta^{op}+10\right)$ is a switching or interpolation function that serves to blend the molar Gibb's free energies, $G^{\beta ,\alpha }$ (units of E/mol), between each phase. To convert the molar Gibb's free energy to an energy density, the quantities are divided by the molar volume, $V_{m}$ (units of $L^{3}/mol$). The composition variables $X^{Al,\alpha /\beta }=X^{Al,\alpha /\beta }\left(x_{i},t\right)$ and $X^{V,\alpha /\beta }=X^{V,\alpha /\beta }\left(x_{i},t\right)$ are used to track the distribution of aluminum and vanadium within the ⍺- and β-phases such that the total composition within the computational domain is calculated using Equation (12) and Equation (13) [21]:

(12) $$X^{Al}\left(x_{i},t\right)=\left(1-h\left(\eta^{op}\right)\right)X^{Al,\beta }+h\left(\eta^{op}\right)X^{Al,\alpha }$$

(13) $$X^{V}\left(x_{i},t\right)=\left(1-h\left(\eta^{op}\right)\right)X^{V,\beta }+h\left(\eta^{op}\right)X^{V,\alpha }$$

Here, we take advantage of the conservation of mass (i.e., $X^{Ti,\alpha /\beta }=1-X^{Al,\alpha /\beta }-X^{V,\alpha /\beta }$) to reduce the number of composition variables required. Point-wise equality of the chemical potential relative to titanium is assured via the constraints defined by Equation (14) and Equation (15):

(14) $$\frac{\partial G^{\alpha }}{\partial X^{Al,\alpha }}=\frac{\partial G^{\beta }}{\partial X^{Al,\beta }}=\mu^{Al}-\mu^{Ti}$$

(15) $$\frac{\partial G^{\alpha }}{\partial X^{V,\alpha }}=\frac{\partial G^{\beta }}{\partial X^{V,\beta }}=\mu^{V}-\mu^{Ti}$$

The evolution of the conserved composition fields is governed by Equation (16) [6,21,22]:

(16) $$\frac{\partial X^{Al,V}}{\partial t}=\nabla_{i}\left(M^{Al,V}\nabla_{i}\left[\frac{\delta F^{\beta \to \alpha /\beta }}{\delta X^{Al,V}}\right]\right)$$

where the term inside of [⋅] represents the functional derivative of the global free energy. The conserved mobility for aluminum and vanadium, $M^{Al,V}$, is in general a tensor that is phase-dependent. This mobility can be modeled using the CALPHAD method or an Arrhenius type equation [9,10]. Here, the diffusion coefficients provided in Ref. [23] (i.e., Equation (17) and Equation (18)) are leveraged:

(17) $$D^{\alpha /\beta ,Al}=1.2\cdot 10^{5}\;\exp\left(-18040/T\right)$$

(18) $$D^{\alpha /\beta ,V}=10^{5}\;\exp\left(-17460/T\right)$$

where the diffusion coefficients are units of μm^2^/s. The mobilities (units of $L^{5}/tE$) can then be calculated using Equation (19) and Equation (20) [6,22]:

(19) $$M^{Al,V}=\frac{D^{\alpha /\beta ;Al,V}}{f_{cc}}$$

(20) $$f_{cc}^{Al,V}=\frac{\frac{\partial^{2}G^{\alpha }}{\partial {\left(X^{Al,V}\right)}^{2}}\frac{\partial^{2}G^{\beta }}{\partial {\left(X^{Al,V}\right)}^{2}}}{\left(1-h\left(\eta \right)\right)\frac{\partial^{2}G^{\beta }}{\partial {\left(X^{Al,V}\right)}^{2}}+\left(h\left(\eta \right)\right)\frac{\partial^{2}G^{\alpha }}{\partial {\left(X^{Al,V}\right)}^{2}}}$$

The evolution of the non-conserved order parameters is governed by the Allen-Cahn equation (Equation (21)) [22]:

(21) $$\frac{\partial \eta }{\partial t}=-M_{\eta }\left[\frac{\delta F^{\beta \to \alpha /\beta }}{\delta \eta }\right]$$

where the non-conserved mobility (units of $L^{3}/Et$) is defined by Equation (22):

(22) $$M_{\eta }=3\cdot 10^{-35}\;\exp\left(0.0768T\right)$$

which was determined by setting $M_{\eta }$ to different values at different temperatures and ensuring that the values were high enough such that the evolution was diffusion-controlled (as opposed to “diffusionless” or martensitic) [24]. That is, ⍺-lath growth was parabolic (i.e., $L_{\alpha }\propto \sqrt{t}$).

The governing equations are also solved using MOOSE. The size of the domain was assumed to be 10 μm. The domain was discretized into 500 line elements ensuring at least 10 elements through the ⍺/β interface. The left and right sides of the domain are set to Neumann boundary conditions for each of the nonlinear variables where the flux is assumed to be zero.

#### 2.2.3 CALPHAD: thermodynamic modeling of Ti-6AL-4V

The chemical free energy for the phase field model is constructed using the molar Gibb's free energies for the ⍺- and β-phases. The Gibb's free energies are determined using the National Institute of Materials Science (NIMS) thermodynamic database [7,8] and the CALPHAD formalisms. Here, we treat the liquid, ⍺-, and β-phases as substitutional solution phases (i.e., homogenous mixtures of elements) whereby the molar Gibb's free energies are defined in Equation (23) through Equation (27) [25]:

(23) $$G^{\varphi }=G^{\varphi ,ref}+G^{\varphi ,cfg}+G^{\varphi ,exs}$$

(24) $$G^{\varphi ,ref}=\sum_{k=Al,Ti,V}X^{k}G^{k,\varphi }$$

(25) $$G^{\varphi ,cfg}=RT\sum_{k=Al,Ti,V}X^{k}\;\ln\left(X^{k}\right)$$

(26) $$G^{\varphi ,exs}=\sum_{\begin{array}{c} r=Al,Ti,V\\ s\neq r \end{array}}X^{r}X^{s}\sum_{k=0}^{N_{L}}L_{r,s}^{\varphi ,k}{\left(X^{r}-X^{s}\right)}^{k}+\sum_{\begin{array}{c} r,s,t=Al,Ti,V\\ r\neq s\neq t \end{array}}X^{r}X^{s}X^{t}\sum_{v=Al,Ti,V}L_{v}^{\varphi }V_{v}$$

(27) $$V_{v}=X^{V}+\frac{1}{3}\;\left(1-\sum_{p=Al,Ti,V}X^{p}\right)$$

where $G^{\varphi ,ref}$, $G^{\varphi ,cfg}$, and $G^{\varphi ,exs}$ represent the reference, configurational, and excess contributions to the total free energy for each phase φ:{liquid,α(HCP_A3),β(BCC_A2)}. The reference Gibb's free energies (i.e., $G^{\varphi ,ref}$) are determined from the Scientific Group Thermodata Europe (SGTE) data for pure elements. This is the Gibb's free energy for each pure component (i.e., k= Al, Ti, V) relative to their stable phase (i.e., $G^{k,\varphi }$) [7,8,26]. The summation of these functions weighted by the mole fractions of the Ti–6Al–4V alloy gives the reference molar Gibb's free energy at a given temperature T.

The configurational contribution (i.e., $G^{\varphi ,cfg}$) is related to the probability that the interstitial or substitutional sites of the HCP_A3_ and BCC_A2_ crystal structures of the solvent (titanium) are occupied by either solute atoms (i.e., aluminum; vanadium) or a vacancy. These sites are associated with a “sublattice” in the Compound Energy Formalism (CEF) [9,10,27]. Within this formalism, the HCP_A3_ and BCC_A2_ phases are modeled as consisting of two sublattices (a,b) with Ti, Al, and V existing on sublattice a and vacancies (Va) existing on sublattice b indicating substitutional phases. This is represented as $HCP\_A3:{\left(Ti,Al,V\right)}_{a}{\left(Va\right)}_{b}={\left(Ti,Al,V\right)}_{3}{\left(Va\right)}_{1}$ and $BCC\_A2:{\left(Ti,Al,V\right)}_{1}{\left(Va\right)}_{0.5}$. The values for a and b indicate a stoichiometric balance between each of the sites and are used as weights in the configurational free energy [9,10,27].

Finally, the excess Gibb's free energy (i.e., $G^{\varphi ,exs}$) is used to account for the interactions between each of the constituents on the sublattice and is determined using interaction parameters, $L_{r,s,t}^{\varphi ,k}$, which are fit to experimental data using Redlich-Kister polynomials and are listed in the NIMS database with the following convention: $L_{r,s,t}^{\varphi ,k}=G\left(\varphi ,r,s,t:VA:k\right)$ [9,10].

**Fig. 5:**
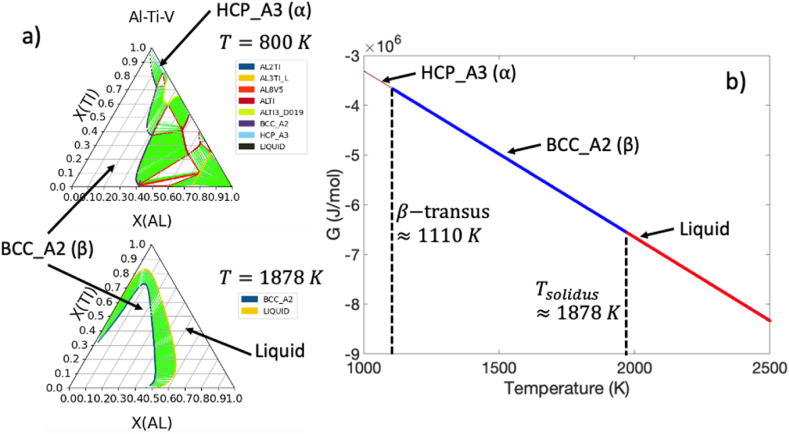
a) Predicted phase diagrams for the Al–Ti–V ternary system at 800 K (top) and 1878 K (bottom); b) Cross-section through the Gibb's free energy surface at the approximate equilibrium composition for Ti–6Al–4V with estimated solidus (1878 K) and β-transus (1110 K) temperatures.

After combining this information into a Python script which leverages pycalphad [28], the predicted ternary phase diagrams at 800 K and 1878 K as well as the predicted β-transus temperature (i.e., $T_{\beta }\approx 1110\;K$) and solidus temperature (i.e., $T_{solidus}\approx 1878\;K$) are shown in Fig. 5a and b, respectively. These values approximately match the experimentally-determined physical values for Ti–6Al–4V [2,3,24]. Therefore, the Gibb's free energy constructed for this alloy can be used within the phase field model.

## 3 Results

### 3.1 Prior-β grain widths

**Fig. 6:**
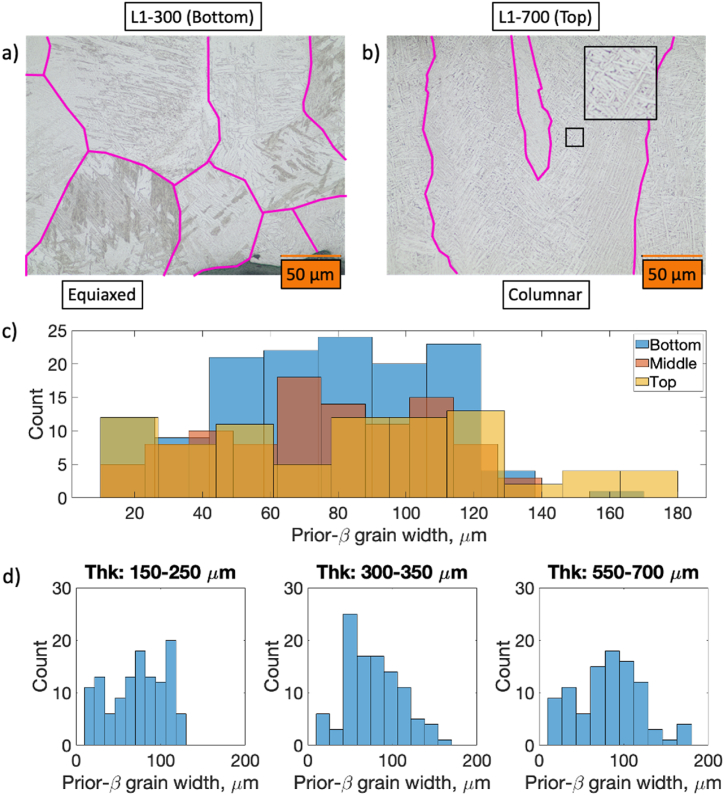
Optical micrographs detailing equiaxed β grains at bottom of specimen L3 (300 μm thick; a) and columnar β grains located near the top of specimen L1 (700 μm thick; b). Prior-β grain width for all specimens separated by through-thickness location (c) and specimen thickness (d).

Example optical micrographs of the L1 specimen at the bottom (15%; thickness: 0.30 mm) and top (85%; thickness: 0.70 mm) locations are shown in Fig. 6a and b, respectively. For clarity, the prior-β grain boundaries are also highlighted in these figures. The micrographs show that the prior-β grains are predominantly columnar with few equiaxed grains located near the bottom of the specimens. Many of the beta grains appear tapered with a positive correlation between the increase in grain width and increases in specimen thickness. These observations are corroborated by the prior-β width distributions provided in Fig. 6c and d. Fig. 6c shows prior-β width measurements collected by thickness location (i.e., bottom; middle; top).

Assuming a normal distribution, the mean and standard deviation for the β grain widths at the bottom, middle, and top locations are 75.6 ± 31.2 μm, 76.02 ± 30.4 μm, and 81.2 ± 44.2 μm, respectively. Likewise, Fig. 6b shows the width distributions separated according to specimen thickness. The mean and standard deviation for thicknesses of 0.15–0.25 mm, 0.30–0.35 mm, and 0.55–0.70 mm are 72.2 ± 32.6 μm, 77.9 ± 33.02 μm, and 82.7 ± 38.6 μm, respectively. For both sets of data, lower thicknesses and regions closer to the build plate are associated with lower means (i.e., thinner prior-β) and standard deviations (i.e., lower deviations in width) than for regions further from the build plate.

### 3.2 ⍺-Lath thicknesses

**Fig. 7:**
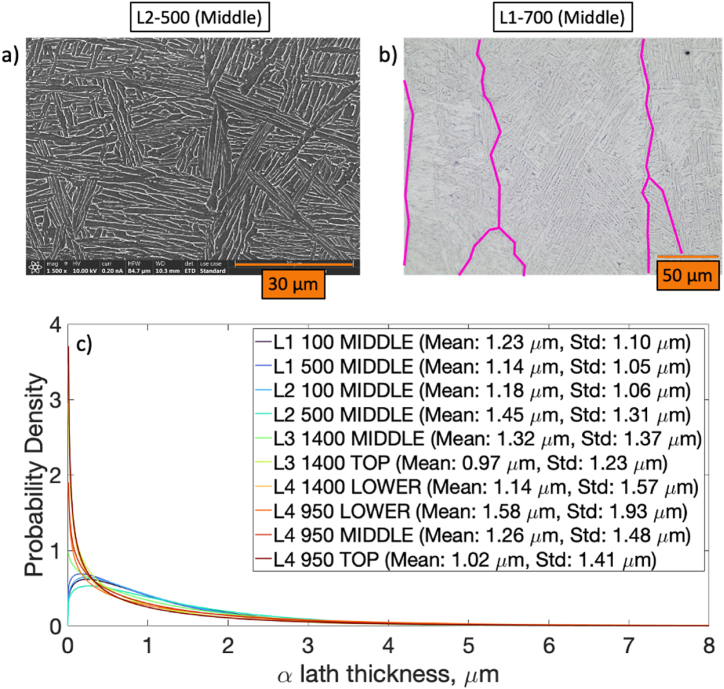
a) Scanning electron micrograph detailing ⍺ laths (dark) and β ribs (light) for middle of L2 specimen (500 μm thickness); b) Optical micrograph of L1-700 specimen highlighting the prior-β grains and typical lamellar ⍺-lath structure; c) Probability density for the ⍺ lath thickness for the L1 through L4 specimens with mean and standard deviations provided in the legend.

Example optical and scanning electron micrographs which detail the Widmanstätten (i.e., “Thomson” or “basket-weave”) ⍺-lath structures are provided in Fig. 7a and b, respectively. The dark regions of the scanning electron micrograph detail the ⍺-laths while the light regions show the β-ribs typical of additively manufactured Ti–6Al–4V [[29], [30], [31]]. Also shown in Fig. 7c is the ⍺-lath thickness Gamma distributions with mean and standard deviations provided in the legend organized by specimen thickness and location. From the data, there does not appear to be an appreciable correlation between ⍺-lath thickness, measurement location, or specimen thickness. How these values correlate with the numerical models will be discussed later.

### 3.3 Phase fractions

**Fig. 8:**
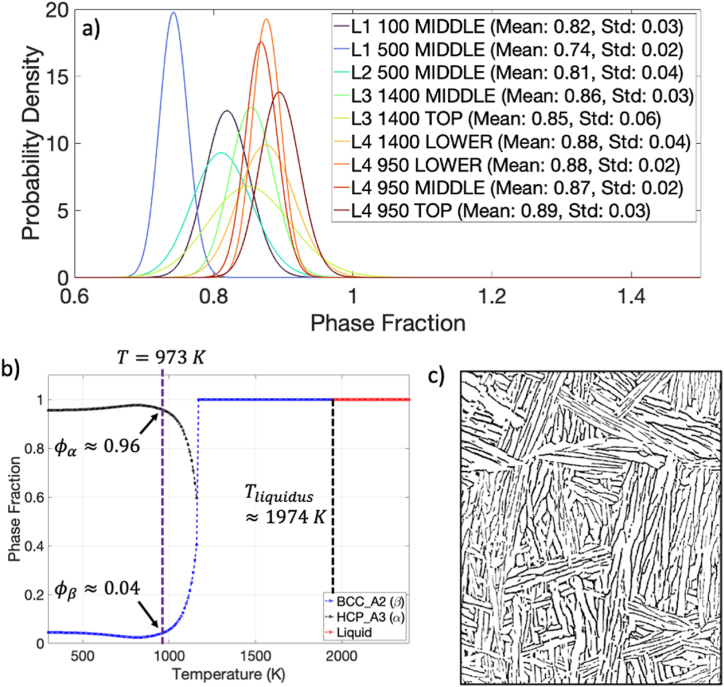
a) The probability densities for the phase fraction for each specimen with mean and standard deviations provided in the legend; b) Calculated phase fraction using the Ti–6Al–4V thermodynamic databases and CALPHAD approach; c) An example detailing the phase fraction where the dark regions represent the β-ribs and the light regions the ⍺-laths.

Finally, the distribution for the phase fractions are provided in Fig. 8a. The mean and standard deviation for the phase fractions for each specimen is provided in the legend. The global mean value is 84% ⍺-phase, 16% β-phase. Also provided in Fig. 8b is the phase fraction calculated using the CALPHAD approach. Below a preheat temperature of 973 K, the CALPHAD method predicts a phase fraction of 96% ⍺-phase, 4% β-phase. These results are comparable to the results of [[29], [30], [31]]. For reference, an example of the Ti–6Al–4V microstructure is shown in Fig. 8c where the dark regions represent the β-ribs and the light regions the ⍺-laths.

### 3.4 Specimen thickness; beam penetration depth

**Fig. 9:**
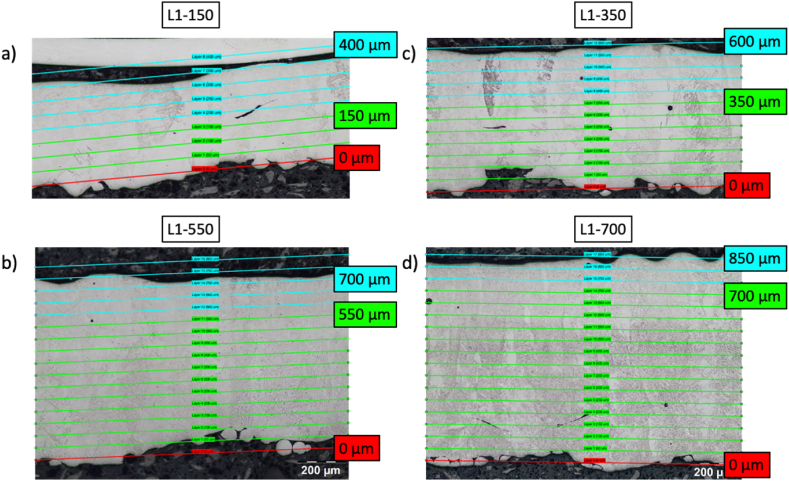
Optical micrographs for: a) L1-150; b) L1-550; c) L1-350; and d) L1-700 specimens. The micrographs show the variation in the nominal (as-designed) specimen thickness [green] and the additional layers [blue] as measured from a datum plane [red]. The results show an additional thickness of 150–250 μm (3–5 layers) which correlates to the beam penetration depth. (For interpretation of the references to color in this figure legend, the reader is referred to the Web version of this article.)

The cross-sections shown in Fig. 9 show the correlation between as-built specimen thickness, nominal layer height, and the nominal CAD geometry shown in green for the L1 specimen (thicknesses: 0.15 mm [Fig. 9a], 0.35 mm [Fig. 9c], 0.55 mm [Fig. 9b], and 0.70 mm [Fig. 9d]). Any layers beyond the nominal CAD geometry are shown in blue. In general, the as-built specimen thickness is larger than the nominal CAD geometry. The increase in thickness is approximately 150–250 μm (i.e., 3–5 layers) which is indicative of the electron beam penetration depth.

**Fig. 10:**
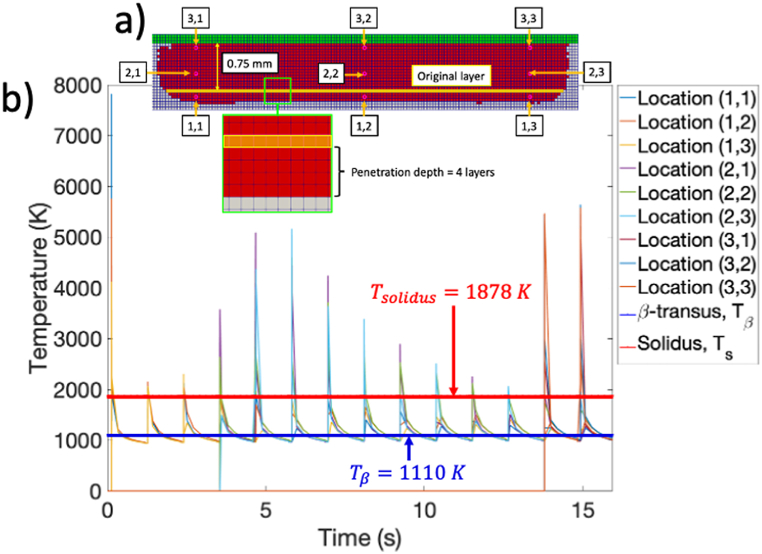
a) Overview of the temperature probe locations (single elements) used to extract the thermal history (b) throughout simulated build process. Note that the results are somewhat misleading as the cooldown step was truncated to reduce computational expense.

Results for the numerical model are shown in Fig. 10a where a penetration depth of 4 layers was measured relative to the original deposited layer which correlates well with the experimental data. Also shown in Fig. 10b are locations where thermal history was extracted during the layer-by-layer process. These locations were selected to correlate with the locations (i.e., 15%; 50%; 85%) shown in Fig. 10a. The spacing between these elements is 7–8 layers. The thermal history for the probes is shown in Fig. 10b relative to CALPHAD-predicted solidus ($T_{solidus}$) and β-transus ($T_{\beta }$) temperatures. Note that the large spikes in temperature are because application of the heat source is instantaneous (as opposed to ramped), which results in a large jump in the temperature (i.e., 7800 K for earlier layers). Also note that the results for Fig. 10b are somewhat misleading as it implies that most of the processing time is spent between the solidus and β-transus temperature. However, the reader is reminded that the timescale for the cooldown step was reduced from an average of 8 min between layers as measured in the logfile to 1 s. Nevertheless, note that for each layer, the temperature of each element exceeds the β-transus indicating dissociation of the ⍺-phase.

**Fig. 11:**
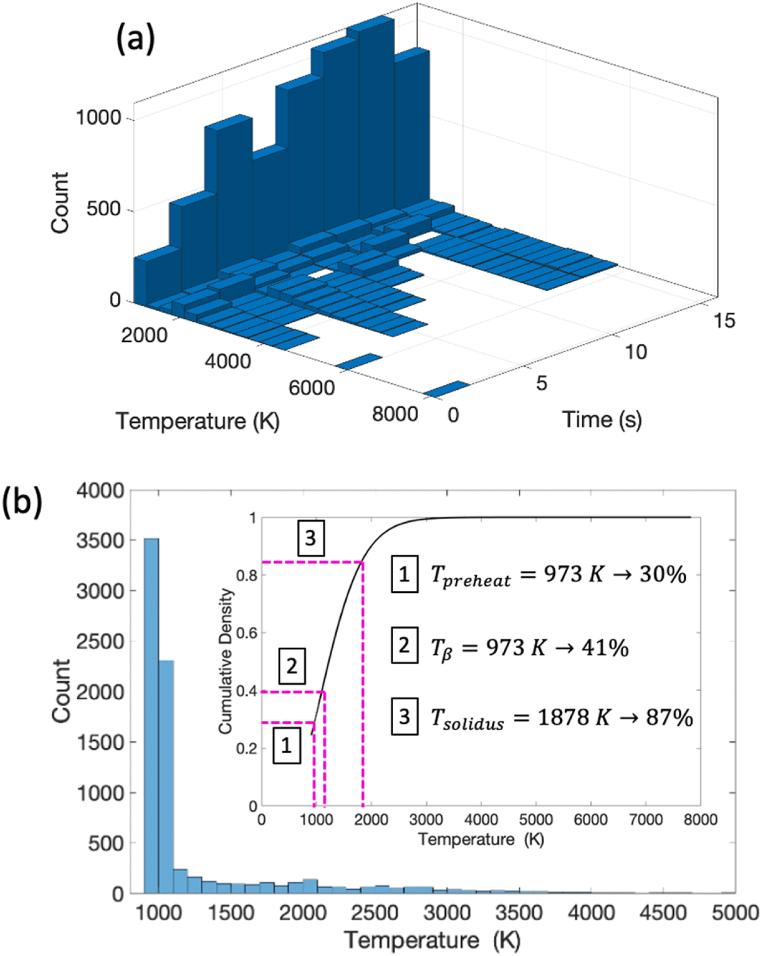
a) Bivariate distribution showing the time temperature data for the nine data points; b) Cumulative density collapsed along the time axis. Data shows that for the simulation, approximately 41% of the time the elements are below the β-transus.

This information is also reflected in the bivariate distribution shown in Fig. 11a which shows the relationship between temperature and simulation time for the locations considered. This shows that, during deposition of subsequent layers, most of the time is spent below the solidus temperature. This is especially true for the lower elements (i.e., Locations: (1,1); (1,2); (1,3) in Fig. 10a). This is expected since the penetration depth of the beam was estimated to be 4 layers. Fig. 11b shows the cumulative density function of the temperature bivariate data collapsed along the time axis. For the simulation duration considered, the cumulative density function shows that 41% of the time, the material is below the β-transus temperature (i.e., ⍺ is nucleating or growing), while 59% of the time, the material is above the β-transus temperature (i.e., ⍺ has dissociated/not nucleating or growing). Note that for more realistic simulation scales (i.e., closer to the time scales for the manufacturing process), it would be expected that the slope of the cumulative density function near the inflection point would increase, resulting in a larger amount of time being spent below the β-transus temperature near the preheat temperature. This indicates that ⍺-lath growth is dominated by coarsening at the preheat temperature.

## 4 Discussion

### 4.1 Melt→β-phase transformation

Per the results provided in Fig. 10, Fig. 11, there are three temperature bands of interest: 1) Above the solidus temperature (i.e., β-phase melting/dissociation); 2) Between the solidus and β-transus temperature (i.e., β-phase formation); and 3) Below the β-transus temperature (i.e., ⍺-phase formation). This last band is the only band where the ⍺ history is not annihilated. Therefore, microstructural formation, and hence the mechanical performance of the as-built product, during the EBM of Ti–6Al–4V is dominated by the time history of the following phase transformations: 1) Melt → β-phase; and 2) β→⍺/β. For the first phase transformation, we utilize the thermal history data provided above and, for comparison purposes, a modified version of the Rosenthal equation [32]. The original Rosenthal equation satisfies Equation (28):

(28) $$\nabla \cdot \left(\nabla T\left(x,y,z,t\right)\right)=\frac{\rho c_{p}}{k_{c}}\frac{\partial T\left(x,y,z,t\right)}{\partial t}$$

which has a solution defined in Equation (29) through Equation (30):

(29) $$R=\sqrt{x^{2}+y^{2}+z^{2}}$$

(30) $$T-T_{preheat}=\frac{\eta P_{b}}{2\pi k_{c}R}\exp\left[-\frac{v_{b}\left(x+R\right)}{2\alpha_{d}}\right]$$

This solution can be used to approximate the temperature field semi-local to the electron beam heat source. It is important to recognize that the material density (ρ); specific heat ($c_{p}$); thermal conductivity ($k_{c}$); and thermal diffusivity ($\alpha_{d}$) are assumed independent of temperature. Furthermore, although temperature is assumed to be time-dependent in the original differential equation, time does not appear in the final solution. Therefore, the Rosenthal equation is a steady-state equation and cannot account for heat source acceleration and thus any changes in the melt pool geometry associated with such movements. An unfortunate consequence of the Rosenthal equation is that it tends to overpredict the thermal gradients near the origin of the beam since by Equation (31):

(31) $$\lim_{R\to 0}\left(T-T_{preheat}\right)=\lim_{R\to 0}\left(\frac{\eta P_{b}}{2\pi k_{c}R}\exp\left[-\frac{v_{b}\left(x+R\right)}{2\alpha_{d}}\right]\right)\to \infty$$

However, intuitively we know that this is a physical impossibility otherwise the beam would burn through the start plate. Instead, temperature histories local to the beam have been simulated [17,[30], [31], [32]] and measured [33] closer to approximately 3500 K. The differences in the predicted temperature histories are likely due to the fact that the Rosenthal equation neglects convective, radiative, and evaporative effects local to the melt pool. Nevertheless, the usefulness of the Rosenthal equation is that it is numerically inexpensive and has been shown to be reasonably accurate far from the melt pool. With this being the case, we should like to reduce the effects of the singularity near the beam origin and replicate temperatures closer to those measured.

In this case, we take a naïve approach and implement a simple mathematical “trick”. The hyperbolic tangent function (i.e., “tanh”) can be used as a numerical “switch” to transition between regions far from the beam origin (i.e., which we want to keep) and local to the beam origin (i.e., which we want to replace). This is accomplished by separating the value, R, in the original Rosenthal equation with two different values defined using Equation (32) through Equation (37):

(32) $$R_{1}=R=\sqrt{x^{2}+y^{2}+z^{2}}$$

(33) $$R_{\max}=\frac{\eta P_{b}\beta_{MR}}{2\pi k_{c}T_{\max}\;}\exp\left[-\frac{v_{b}\left(x+R_{1}\right)}{2\alpha_{d}}\right]$$

(34) $$S_{x}=\left(0.5+0.5\;\tanh\left(\gamma_{w}x\right)\right)\left(0.5+0.5\;\tanh\left(-\gamma_{w}x\right)\right)$$

(35) $$S_{y}=\left(0.5+0.5\;\tanh\left(\gamma_{w}y\right)\right)\left(0.5+0.5\;\tanh\left(-\gamma_{w}y\right)\right)$$

(36) $$S_{z}=\left(0.5+0.5\;\tanh\left(\gamma_{w}z\right)\right)\left(0.5+0.5\;\tanh\left(-\gamma_{w}z\right)\right)$$

(37) $$R_{2}=S_{x}S_{y}S_{z}R_{\max}+R_{1}$$

where $T_{\max}$ is the maximum temperature; $\beta_{MR}$ is a tuning parameter; and $S_{x\to z}$ are the switching functions used to transition between far-field regions and regions close to the singularity with transition thickness, γ. The modified Rosenthal equation then becomes (Equation (38)):

(38) $$T-T_{preheat}=\frac{\eta P_{b}}{2\pi k_{c}R_{2}}\exp\left[-\frac{v_{b}\left(x+R_{1}\right)}{2\alpha_{d}}\right]$$

**Fig. 12:**
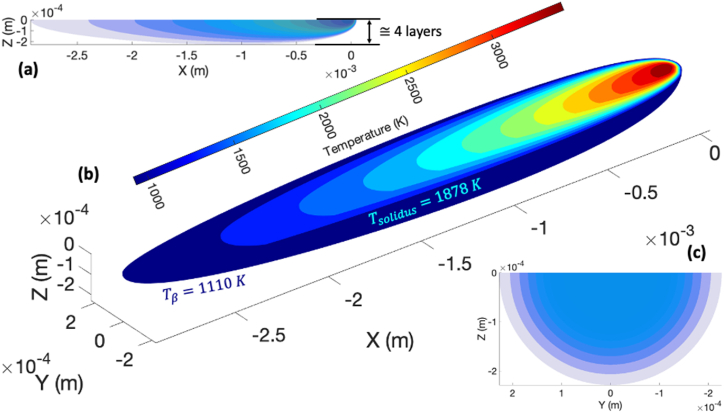
Temperature distribution for the modified Rosenthal equation as viewed from: a) XZ plane; b) isometric; and c) YZ plane. The maximum temperature is approximately 3500 K.

At this point it is important to remind the reader that this numerical trick does not necessarily satisfy the original partial differential equation near the singularity. However, the value in using the Rosenthal equation is not in trying to resolve the details near the singularity but rather understanding the far-field temperature distributions which *will* satisfy the partial differential equation within numerical precision. There are two benefits of this modification. First, the temperature distributions near the beam origin are more representative of those measured and calculated using more advanced methods. Second, removing the singularity makes some of the analyses more straightforward and convenient. The temperature distribution for the modified Rosenthal (MR) equation is shown in Fig. 12a and. b, and Fig. 12c which show the XZ-plane, isometric, and YZ-plane projections, respectively.

With the temperature distributions approximated from the MR equation, the temperature gradient components as well as their magnitude can be calculated using Equation (39) and Equation (40):

(39) $$\nabla T=\left(\frac{\partial T}{\partial x},\frac{\partial T}{\partial y},\frac{\partial T}{\partial z}\right)\approx \left(\frac{\Delta T}{\Delta x},\frac{\Delta T}{\Delta y},\frac{\Delta T}{\Delta z}\right)$$

(40) $$\left\|\nabla T\right\|=\sqrt{{\left(\frac{\partial T}{\partial x}\right)}^{2}+{\left(\frac{\partial T}{\partial y}\right)}^{2}+{\left(\frac{\partial T}{\partial z}\right)}^{2}\;}\approx \sqrt{{\left(\frac{\Delta T}{\Delta x}\right)}^{2}+{\left(\frac{\Delta T}{\Delta y}\right)}^{2}+{\left(\frac{\Delta T}{\Delta z}\right)}^{2}}$$

A comparison of the temperature gradient magnitudes of the original Rosenthal (OR) and MR equations reveals that the calculated temperature gradients for the OR equation are much larger (∼10000⨉) greater than those calculated using the MR (i.e., 10^11^ K/m vs. 10^7^ K/m).

**Fig. 13:**
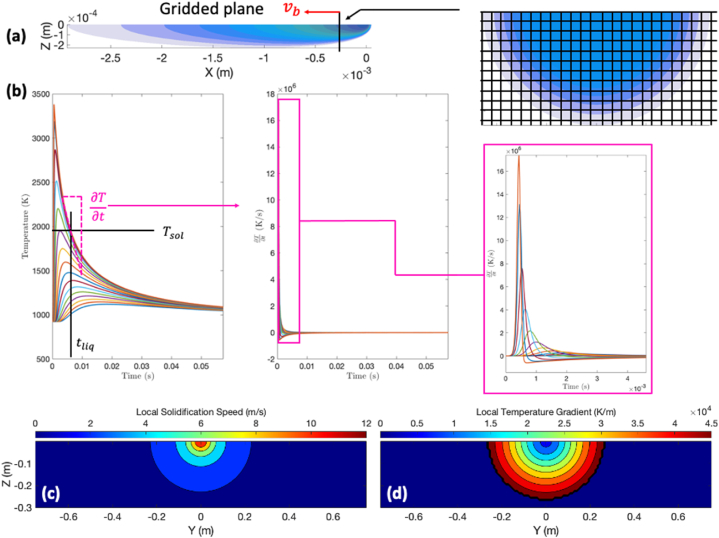
a) Three-dimensional temperature contour plots of the modified Rosenthal equation; b) Plots of temperature as a function of time used to approximate cooling rate at the solidus temperature; Contour plots of the local solidification velocity (c) and temperature gradients (d).

We would like to use this information to approximate the cooling rate, $\frac{\partial T}{\partial t}$, and solidification velocity, $R_{s}$, which will allow us to predict the microstructure morphology that is formed during solidification (i.e., columnar vs. equiaxed). At steady-state conditions, the cooling rate is approximated by first partitioning the YZ-plane into a point grid. Next, the point grid is translated along the longitudinal axis of the beam, opposite to the direction of beam travel, at a speed $v_{b}$. The instantaneous temperature within the grid is tracked over the time taken for the grid to traverse the length of the beam which can be approximated by the location of the solidus isotherm. The method as well as the temperature profiles that result are shown in Fig. 13a and b. Next, the thermal gradient local to the “pierce point” of the solidus isotherm and the cooling rate at this point can be used to estimate the solidification velocity via Equation (41) [34]:

(41) $$R_{s}\approx \frac{\partial T/\partial t}{G}$$

A plot of the solidification velocity and temperature gradient at the pierce point of the solidus isotherm is shown in Fig. 13c. Anything outside of the semicircles is considered solid material.

At first glance, the information contained in Fig. 13c and d may seem counterintuitive. For instance, we know that regions near the beam origin are associated with a high thermal gradient and, perhaps, a high solidification velocity. However, the results do not show this. This apparent contradiction can be explained by reminding the reader that solidification is not necessarily initiated local to the beam origin and instead depends on the location of the liquidus and solidus isotherms. As the beam traverses through the material, different points throughout the material will be at different temperatures depending on their position within the temperature field defined by the MR equation. Consequently, some material points will melt while others will not. Furthermore, some material points will experience different local thermal gradients depending on their locations within the temperature field. The time spent at these locations as they relate to the beam velocity as well as the material properties affects the temperature rate which governs the solidification velocity. Therefore, the information provided Fig. 13 can be used to estimate the microstructural texture based on the relationships between the temperature gradient and the solidification velocity as determined in Refs. [2,3] for Ti–6Al–4V.

**Fig. 14:**
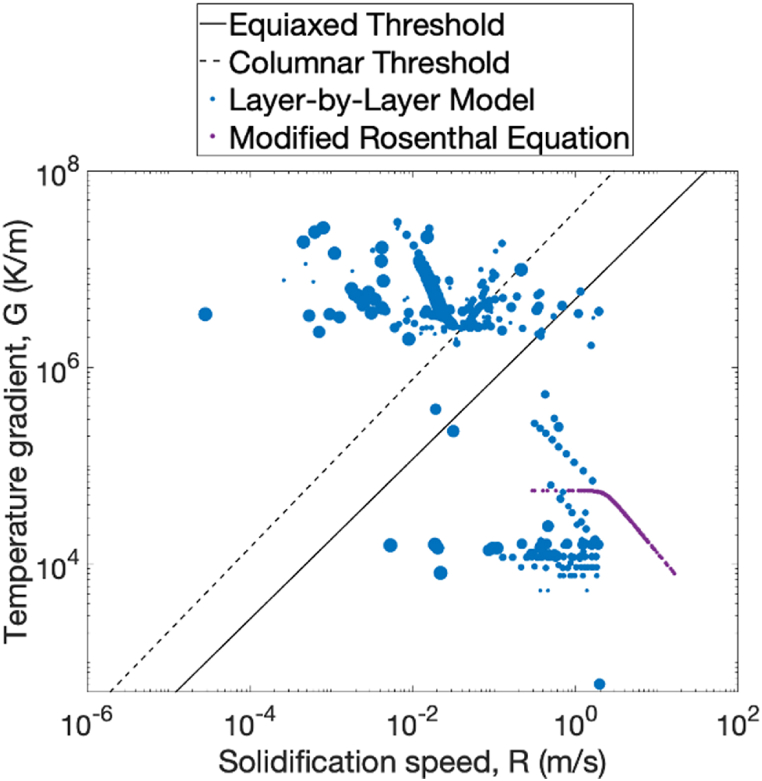
Solidification speed and temperature gradient. This information is used to predict the microstructure texture/morphology via the solidification map using the data provided in Refs. [2,3].

As shown in Fig. 14, the model predicts that the microstructure in the wake of the electron beam is predominantly equiaxed. Therefore, the mixed columnar-equiaxed grains are a result of the layer-by-layer process. This is in contrast to predictions of the OR equation which show a predominantly columnar structure likely due to the much higher thermal gradients calculated as a result of the singularity at the beam origin. Locations of the isotherms are also affected and result in overestimations of the solidification velocities at similar beam speeds [1]. Furthermore, while the OR model tends to predict columnar microstructures, advanced computation fluid dynamics (CFD) models which account for the radiative and evaporative heat losses at the melt pool show lower temperatures and hence lower thermal gradients thereby predicting mixed columnar-equiaxed grains [17]. The 2D layer-by-layer finite element model considered here also predicts this as shown in Fig. 14 where the size of each marker or point is correlated with time. That is, earlier layers have smaller points than later layers which have larger points. From this data, it appears that later layers are associated with higher temperature gradients and lower estimates for solidification speed leading to a transition from equiaxed to columnar grains. This change in temperature gradients and solidification speeds could be one explanation as to why equiaxed grains are shown at the bottom of the L1-300 specimen in Fig. 6a.

### 4.2 β→⍺/β transformation

Fig. 10b shows that for the specimen thicknesses considered (i.e., both experimental and numerical; 0.70 mm), each layer exceeds the β-transus. Therefore, even after the final layer of material is deposited, the ⍺-phase located at the bottom layer dissociates. A few conclusions can be made from this observation: 1) For the specimen thicknesses considered, any through-thickness variation in the ⍺-lath thickness is due to through-thickness cooling after the final layer has been deposited; 2) There is a critical layer thickness where the ⍺-phase does not dissociate and, instead, subsequent heat input results in temperature increases and commensurate increases in ⍺-phase coarsening kinetics.

We can estimate this critical layer thickness as follows: For the phase transformation of β→⍺/β, we note that a preheat temperature of 973 K and a β-transus of 1110 K results in a temperature change, ΔT, of 137 K required to cause dissociation of the ⍺-phase. Specifically, we are interested in the height of material in which the β-transus is no longer exceeded. Consider the amount of energy required to raise the temperature of a mass of material described by Equation (42):

(42) $$q=mc_{p}\Delta T$$

where q is the amount of heat input; m is the material mass; $c_{p}$ is the specific heat; and ΔT is the change in temperature. This relationship can be modified to give Equation (43):

(43) $${\dot{Q}}_{b}\Delta t=\frac{{\dot{Q}}_{b}D_{b}}{v_{b}}=\rho Vc_{p}\Delta T$$

where ${\dot{Q}}_{b}$ is the rate of heat input (i.e., beam power); Δt is the residence time of the beam of diameter $D_{b}$ and velocity $v_{b}$; ρ is the mass density; and V is the material volume. We consider a material volume that is a cylinder with height, $d_{p}$, below the beam defined by Equation (44):

(44) $$V=\frac{\pi }{4}D_{b}^{2}d_{p}$$

We can substitute this into the relationship above and solve for $d_{p}$ to give Equation (45):

(45) $$d_{p}=\frac{4{\dot{Q}}_{b}}{\pi D_{b}v_{b}\rho \Delta Tc_{p}}$$

From this, the height is estimated to be $d_{p}\approx 11\;mm$ or 220 layers which is approximately 10X the thickness of the specimens considered here. Therefore, for the thin Step-Ramp specimens, the β-transus will be exceeded for each layer. This indicates that ⍺-phase growth is dominated by nucleation and coarsening after the final layer is deposited. While a somewhat crude estimate, this result is corroborated by the experiments of Ghods et al. [29]. In these experiments, Ghods et al. built two specimens: One “staircase” configuration (11 X 11 × 10 mm) and one “pyramid” configuration (24 mm tall with a 12 mm radius). For each configuration, the ⍺-lath thickness was measured near at bottom (0.5 mm; 2 mm), middle (5.5 mm, 10 mm), and top (10.5 mm, 22 mm) locations similar to the specimens discussed here. The results provided in Ref. [29] show a difference between the ⍺-lath thicknesses measured for the pyramid specimen (where $d_{p}\geq 11\;mm$) at the bottom and middle locations as compared to the top locations while for the staircase specimen, only slight deviations exist ($d_{p}<11\;mm$).

**Fig. 15:**
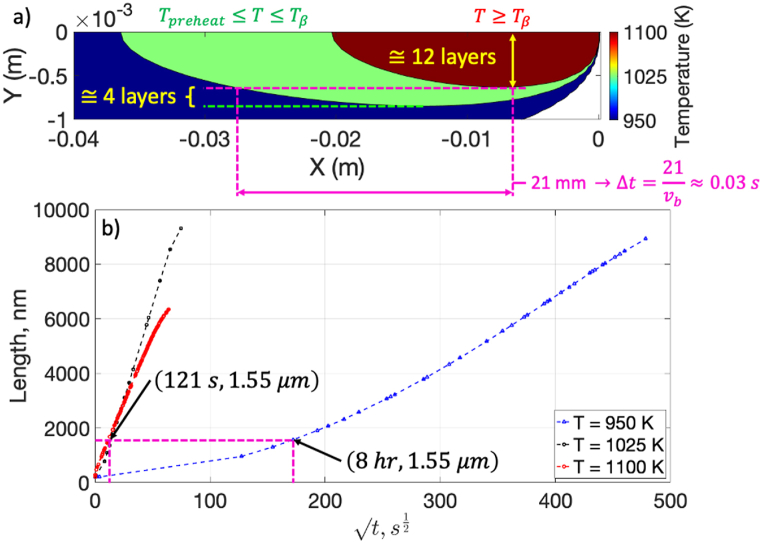
a) Contour plot of temperature along the centerline of the MR equation; plot shows zones that are above the β-transus (no ⍺ formation), between the β-transus and preheat temperature (⍺ formation), and below the preheat temperature (⍺ formation; slow growth kinetics); b) Growth kinetics of the ⍺-lath as predicted by the KKS phase field model for zones shown in a).

These results are also reflected in Fig. 15a which shows the temperature contours for the MR equation for temperatures above the β-transus, temperatures between the preheat temperature and β-transus, and temperatures at the preheat temperature. The temperature zone depth above the β-transus is approximately 12 layers while the zone depth between the preheat and β-transus temperature is approximately 4 layers. Furthermore, the length of this zone is approximately 21 mm which results in a residence time of 0.03 s. The growth kinetics of the ⍺-phase as predicted by the KKS model shows that to achieve an ⍺-lath thickness of 1.55 μm, it would take 121 s at temperatures between the preheat and β-transus temperature and 8 h at the preheat temperatures (Fig. 15b). Note that 8 h is the duration of the original Step-Ramp build and the ⍺-lath thicknesses predicted by the KKS model are corroborated with the mean thicknesses measured experimentally (Fig. 7). Therefore, these results indicate that there are two mechanisms for ⍺-phase coarsening: 1) Coarsening at the preheat temperature; and 2) Thermal cycling due to local energy deposition of subsequent beam passes. This thermal cycling can be used to increase the kinetics of ⍺-phase growth and can be used to tailor the microstructure as has been done by others [35]. Each will have an effect on the local yield strength of the material via Hall-Petch-type relationships [1].

### 4.3 $\alpha^{\prime }$ (martensite) formation

While not explicitly measured in this study, a discussion on the formation of $\alpha^{\prime }$ is warranted. This phase is associated with rapid solidification and cooling from the melt. As such formation of $\alpha^{\prime }$ is classified as a diffusionless martensitic transformation due to alloy element partitioning of aluminum and vanadium during solidification. From Fig. 9, a microstructural gradient can be observed in each of the optical micrographs between the top 3–4 layers (i.e., the beam penetration depth) and the remaining lower layers. Per the results from the layer-by-layer model, each cooling step exceeds the martensitic cooling rate of 525 °C/s indicating the formation of $\alpha^{\prime }$. Results by Al-Bermani et al. investigated phase formation of EBM Ti–6Al–4V specimens using x-ray diffraction (XRD) experiments for 1 mm and 25 mm thick specimens. In general, the group found that the 1 mm thick specimens consisted mostly of $\alpha^{\prime }$ due to martensitic transformations during solidification while the thicker 25 mm specimens resulted in eventual transformation of $\alpha^{\prime }$ to ⍺/β due to the accumulation of additional thermal mass [1]. Similar investigations were performed by Mur et al. in determining hardness vs. time relationships for quenched Ti–6Al–4V specimens. This group found that complete transformation of the $\alpha^{\prime }$ to ⍺/β was only achieved after annealing at 973 K (i.e., the EBM preheat temperature) for approximately 1 h [36].

## 5 Conclusion

The purpose of this work was to investigate and validate the application of numerical models in predicting microstructural formation of thin structures. To study this, four (4) physical specimens were manufactured using an Arcam A2X EBM machine. Each specimen was nominally 20 mm wide and 140 mm long and had thicknesses that varied either uniformly (i.e., “ramped”) or discretely (i.e., “stepped”) between 0.1 and 0.7 mm or 0.2–1.4 mm. Additionally, two finite element models were developed: 1) A macroscale model based on the element birth/death method used to investigate the temperature evolution that occurs during the layer-by-layer build process.; and 2) A mesoscale phase-field model based on the Kim-Kim-Suzuki method used to investigate ⍺-phase growth kinetics. The phase-field model was coupled to a Ti–6Al–4V thermodynamic database using the CALPHAD approach. The results for the models were compared to experimental data collected using optical and scanning electron microscopy. In general, the study found that a critical layer thickness (estimated to be 11 mm) exists whereby the melting of subsequent layers does not result in the dissociation of the ⍺-phase in previous layers. This indicates that the formation of microstructure gradients in thinner specimens is dominated by local temperature gradients, cooling, and coarsening at the preheat temperature after the final layer has been deposited. Furthermore, for thicker specimens that exceed the critical layer thickness, the ⍺-phase does not completely dissociate and is estimated to be a function of the continuous energy deposition and temperature distributions due to conduction and coarsening at the preheat temperature. This reveals that local scanning or energy deposition strategies may be effective at controlling the ⍺-phase provided that the β-transus is not exceeded. This results in higher growth kinetics and subsequent coarsening of the microstructure that will not be achieved within the span of a build at the comparatively lower preheat temperature. The results also indicate that phase-field models and the CALPHAD approach can be effective in predicting the microstructural formation during the EBM process.

## Data availability

The raw/processed data required to reproduce these findings cannot be shared at this time as the data also forms part of an ongoing research project.

## CRediT authorship contribution statement

**Garrett M. Kelley:** Data curation, Formal analysis, Investigation, Software, Validation, Writing – original draft. **M. Ramulu:** Conceptualization, Methodology, Project administration, Resources, Supervision, Writing – review & editing.

## Declaration of competing interest

The authors declare the following financial interests/personal relationships which may be considered as potential competing interests:Ramulu Mamidala reports administrative support was provided by 10.13039/100007812University of Washington. Ramulu Mamidala reports a relationship with 10.13039/100000003The Boeing Company that includes: non-financial support. If there are other authors, they declare that they have no known competing financial interests or personal relationships that could have appeared to influence the work reported in this paper.
